# Plasma levels of miR‐29b and miR‐200b in type 2 diabetic retinopathy

**DOI:** 10.1111/jcmm.14030

**Published:** 2018-11-23

**Authors:** Maria Enoia Dantas da Costa e Silva, Evelise Regina Polina, Daisy Crispim, Renan Cesar Sbruzzi, Daniel Lavinsky, Felipe Mallmann, Nidiane Carla Martinelli, Luis Henrique Canani, Katia Gonçalves dos Santos

**Affiliations:** ^1^ Laboratory of Human Molecular Genetics Universidade Luterana do Brasil (ULBRA) Canoas RS Brazil; ^2^ Endocrine Division Hospital de Clínicas de Porto Alegre (HCPA) Porto Alegre RS Brazil; ^3^ Department of Ophthalmology and Otorhinolaryngology Universidade Federal do Rio Grande do Sul (UFRGS) Porto Alegre RS Brazil; ^4^ Ophthalmology Division Hospital de Clínicas de Porto Alegre (HCPA) Porto Alegre RS Brazil; ^5^ Department of Biology – Sussman Lab San Diego State University San Diego California; ^6^ Department of Internal Medicine Universidade Federal do Rio Grande do Sul (UFRGS) Porto Alegre RS Brazil; ^7^ Cardiology Division Hospital de Clínicas de Porto Alegre (HCPA) Porto Alegre RS Brazil

**Keywords:** circulating levels, diabetic retinopathy, epigenetics, gene expression, microRNA, type 2 diabetes mellitus

## Abstract

MicroRNAs (miRNAs/miRs) are involved in the pathogenesis of diabetes mellitus and its chronic complications, and their circulating levels have emerged as potential biomarkers for the development and progression of diabetes. However, few studies have examined the expression of miRNAs in diabetic retinopathy (DR) in humans. This case‐control study aimed to investigate whether the plasma levels of miR‐29b and miR‐200b are associated with DR in 186 South Brazilians with type 2 diabetes (91 without DR, 46 with non‐proliferative DR and 49 with proliferative DR). We also included 20 healthy blood donors to determine the miRNA expression in the general population. Plasma levels of miR‐29b and miR‐200b were quantified by reverse transcription‐quantitative polymerase chain reaction (RT‐qPCR). Proliferative DR was inversely associated with plasma levels of miR‐29b (unadjusted OR = 0.694, 95% CI: 0.535‐0.900, *P* = 0.006) and miR‐200b (unadjusted OR = 0.797, 95% CI: 0.637‐0.997, *P* = 0.047). However, these associations were lost after controlling for demographic and clinical covariates. In addition, patients with type 2 diabetes had lower miR‐200b levels than blood donors. Our findings reinforce the importance of addressing the role of circulating miRNAs, including miR‐29 and miR‐200b, in DR.

## 1 INTRODUCTION

Diabetic retinopathy (DR) is a neurovascular disorder and the leading cause of visual impairment and blindness among working‐age adults.^1^, ^2^ In addition to vision loss, DR is associated with chronic kidney disease and mortality from cardiovascular disease in diabetes.^1^ Although longer diabetes duration, poor glycemic control and high blood pressure are the major risk factors of DR,^1^, ^2^, ^3^ epidemiological data support the hypothesis of differential genetic susceptibility to this chronic complication.^1^, ^4^ Epigenetic mechanisms, such as DNA methylation, histone post‐translational modifications in chromatin and non‐coding RNAs, mediate the interplay between genetic and environmental risk factors. Persistence of epigenetic changes might contribute to the metabolic memory phenomenon and to the oxidative stress, inflammation and extracellular matrix accumulation,^5^, ^6^ all of which lead to the development of DR.^5^

In this context, several studies have reported abnormal expression of microRNAs (miRNAs/miRs) in retinal cells under hyperglycemic conditions and in murine models of DR.^7^, ^8^, ^9^ MiRNAs are small endogenous non‐coding RNAs that in general silence gene expression at the posttranscriptional level by pairing to specific sequences in the 3’‐untranslated region of their target mRNAs, thereby repressing protein synthesis. MiRNAs regulate cell development and function by modulating the acquisition and maintenance of beta‐cell identity,^10^ cell growth, proliferation, differentiation, apoptosis and metabolism.^11^ Given their high stability and omnipresence in body fluids, several studies have identified the potential of circulating miRNAs as biomarkers of diagnosis, prognosis and management of type 2 diabetes and its vascular complications.^8^, ^12^, ^13^, ^14^ Despite this, the profile of circulating miRNAs in DR has been evaluated only recently.^15^, ^16^, ^17^, ^18^, ^19^, ^20^, ^21^, ^22^, ^23^, ^24^, ^25^

MiR‐29b and miR‐200b were one of the first miRNAs found to be dysregulated in high glucose‐exposed retinal cells and diabetic murines, thereby suggesting their involvement in the development of DR. MiR‐29b exerts anti‐apoptotic and antifibrotic effects on retinal cells^12^, ^26^ and contributes to endothelial function by increasing nitric oxide,^27^ while miR‐200b protects against vascular permeability and angiogenesis. Down‐regulation of miR‐29b results in increased matrix deposition and retinal fibrosis, whereas down‐regulation of miR‐200b results in increased inflammation. MiR‐29b and miR‐200b are down‐regulated in diabetes resulting in increased angiogenesis and neovascularization in retina.^12^ However, to the best of our knowledge, no study to date has assessed the circulating levels of these two miRNAs in type 2 diabetic patients with DR (cases) and without this complication (controls). Therefore, we investigated the association of plasma levels of miR‐29b and miR‐200b with DR in South Brazilians with type 2 diabetes mellitus.

## 2 MATERIALS AND METHODS

### 2.1 Study subjects and data collection

This case‐control study included a total of 186 type 2 diabetic patients and 20 healthy blood donors. Patients were prospectively enrolled from March 2015 to November 2017 in either the Endocrinology (n = 151) or the Ophthalmology (n = 35) Outpatient Clinic of a tertiary care university hospital (Hospital de Clínicas de Porto Alegre – HCPA) in Porto Alegre, the capital of the State of Rio Grande do Sul in Southern Brazil. Type 2 diabetes was diagnosed according to the American Diabetes Association criteria,^28^ and inclusion criteria in the study were age at diagnosis of diabetes ≥30 years, no need of permanent insulin treatment within the first year of diagnosis and no previous episodes of ketoacidosis. Patients underwent a clinical and biochemical evaluation consisting of physical examination and routine laboratory exams, such as glycated hemoglobin, serum creatinine, lipid profile and urinary albumin excretion, which were measured using standard methods.^29^ Glomerular filtration rate was estimated (eGFR) with the CKD‐EPI equation.^30^ Data regarding the age, gender, age at diabetes diagnosis, smoking history, use of medication and presence of arterial hypertension and other comorbidities were obtained from medical records and interview with a structured questionnaire.

Diabetic retinopathy was diagnosed by fundus photography with dilated pupils by a single trained examiner, using a retinograph (Digital Retinal Camera CR‐2, Canon Inc, Tokyo, Japan), and confirmed independently by two ophthalmologists specialized in retina, who were blinded to the patients’ clinical information. Exclusion criteria included any clinical condition that impairs fundus examination, such as severe cataract. Retinopathy was classified according to the worst affected eye, and was graded as absent (no fundus abnormalities), non‐proliferative (NPDR; microaneurysms, hard exudates, retinal hemorrhages and intraretinal microvascular abnormalities) or proliferative (PDR; neovascularization, fibrous tissue or haemorrhage in the vitreous).^31^, ^32^ Patients who had been previously treated with panretinal photocoagulation were also considered as having PDR. In addition, patients without DR had to have a known diabetes duration of at least 5 years. Of the 186 patients with type 2 diabetes included in the study, 91 patients did not have DR, whereas the remaining had NPDR (n = 46) or PDR (n = 49).

We also included 20 healthy blood donors from the Haemotherapy Division of HCPA to determine the plasma levels of miR‐29b and miR‐200b in our general population. They were enrolled between August and December 2017 and data regarding age, gender and skin colour/ethnicity were collected. Blood donors with a known personal and/or first‐degree family history of diabetes were not included in the study, and no additional clinical or laboratory data were collected from them.

This study was approved by the Research Ethics Committees of HCPA (CAAE number 35065914.9.0000.5327) and ULBRA (CAAE number 35065914.9.3001.5349), and was carried out in accordance with the World Medical Association Declaration of Helsinki.^33^ All subjects provided written informed consent prior to the data and blood collection. Skin colour/ethnicity was self‐reported and categorized as white or non‐white (pardo or black).

### 2.2 MiRNA isolation and quantification

Peripheral blood samples were collected in EDTA‐containing tubes and centrifuged at 2500 *g* for 15 minutes at 4°C within 3 hours from collection. Aliquots of plasma samples were frozen at –70°C until RNA isolation and subjected to freeze‐thawing once. No sample used to extract RNA was haemolysed (based on visual inspection). MiRNAs were isolated from 495 μL of plasma using the mirVana PARIS kit following the enrichment protocol for small RNAs (Ambion; Thermo Fisher Scientific, Waltham, MA, USA). After protein denaturation, 5 μL of 10 pmol/μL of synthetic miR‐39 from *Caenorhabditis elegans* (cel‐miR‐39; Qiagen, Valencia, CA, USA) were spiked‐in to plasma samples to control for technical variations throughout the miRNA isolation and quantification. RNA concentration was determined by spectrophotometry (NanoDrop 2000, Thermo Fisher Scientific).

Plasma levels of miR‐29b and miR‐200b were quantified by two‐step reverse transcription‐quantitative polymerase chain reaction (RT‐qPCR) on the StepOnePlus Real‐Time PCR System (Life Technologies; Thermo Fisher Scientific), using either plates or optical tubes from the same manufacturer (Life Technologies). Ten nanograms of the isolated RNA were reverse transcribed in a total volume of 15 μL with a commercial kit according to the manufacturer's protocol (TaqMan^®^ MicroRNA RT; Life Technologies). Reactions were incubated at 16°C for 30 minutes, 42°C for 30 minutes and 85°C for 5 minutes and held at –20°C. Then, 2 μL of the cDNA reaction mixture were amplified in triplicate reactions by qPCR using pre‐designed miRNA assays, containing specific primers and hydrolysis probes (Life Technologies; ID numbers 000200, 000413 and 002251 for cel‐miR‐39‐3p, hsa‐miR‐29b‐3p and hsa‐miR‐200b‐3p respectively). In addition to the cDNA, the 15 μL PCR reaction mixture contained 0.75 μL of the assay, 7.5 μL of universal PCR master mix (no UNG) and 4.75 μL of nuclease‐free water. Amplification reactions were incubated at 95°C for 10 minutes, followed by 45 cycles of 95°C for 15 seconds and 60°C for 1 minute. Fluorescence data collected during the amplification runs were analysed with the StepOne software version 2.3 (Life Technologies). Individual qPCR reactions generating raw C_q_ > 40 were considered as undetectable.

Relative expression levels were estimated using the comparative method (2^–ΔΔCq^)^34^ considering the spiked‐in cel‐miR‐39 as the reference gene and a pool of cDNA samples obtained from nine subjects with type 2 diabetes randomly selected (four without DR, three with NPDR and two with PDR) as the reference sample (calibrator). Fold‐change values were then log2‐transformed for statistical analysis (see Data S1 for expression data).

### 2.3 Statistical analysis

Categorical data are shown as absolute frequency (percentage) and continuous variables are expressed as mean ± SD or as median (25th and 75th percentiles). Data normality was assessed using the Shapiro‐Wilk test. Categorical data were compared between the groups of subjects by the chi‐square test, and continuous data were compared by independent Student *t* test, Mann‐Whitney *U*, one‐way ANOVA or Kruskal‐Wallis, followed by the Tukey or Dunn post hoc analysis, as appropriate. Correlation between both miRNAs was evaluated using the Pearson (*r*) or the Spearman (*r_s_*) correlation coefficient, as indicated by the normality test. Association of the plasma levels of miR‐29b and miR‐200b with PDR was evaluated by logistic regression analysis. Statistical analyses were done using the spss statistical package version 18.0 (SPSS Inc, Chicago, USA), and two‐tailed *P *< 0.05 were considered as statistically significant.

As DR was the primary outcome of our study, power calculations indicated that the number of samples required for detecting an 1.5‐fold change with a statistical power of 90% at a significance level of 0.05 was 96 (48 cases and 48 controls). We used the value of 1.5 based on the assumption that this difference in miRNA expression might have a biological significance.^26^, ^35^ Power calculations were estimated using the WinPEPI (version 11.43) statistical software.^36^

## 3 RESULTS

### 3.1 Demographic and clinical profile of study subjects

The demographic and clinical characteristics of the patients with type 2 diabetes are shown in Table 1. Briefly, patients with PDR were older and more often male, had a longer duration of diabetes and lower body mass index (BMI), were less often hypertensive, had higher levels of serum creatinine and lower eGFR as compared with patients without DR. Moreover, daily insulin use was more frequent among patients with DR than in those without this complication (Table 1). Although blood donors were younger (mean age 45.5 ± 7.5 years) than type 2 diabetic patients (*P* < 0.001), the proportion of males (45%) and non‐white subjects (30%) among blood donors was virtually the same as that observed among patients with type 2 diabetes (44% and 29%, respectively; *P* > 0.999 for both characteristics).

**Table 1:** Demographic and clinical characteristics of patients with type 2 diabetes

Variable	All patients (n = 186)	Without DR (n = 91)	NPDR (n = 46)	PDR (n = 49)	*P* value
Age (y)	61.1 ± 8.2	60.3 ± 8.3^a^	60.0 ± 9.2^a,b^	63.8 ± 6.6^b^	0.032
Male gender (n, %)	82, 44.1	30, 33.0^a^	22, 47.8^a,b^	30, 61.2^b^	0.005
Non‐white (n, %)	53, 28.5	23, 25.3	14, 30.4	16, 32.7	0.618
Diabetes duration (y)	16.0 ± 8.1	14.8 ± 7.7^a^	14.5 ± 7.2^a^	19.7 ± 8.4^b^	<0.001
Glycated haemoglobin (%)	8.4 ± 1.6	8.4 ± 1.6^a^	9.1 ± 1.8^b^	7.9 ± 1.2^a^	0.002
Insulin use (n, %)	125, 67.2	48, 52.7^a^	38, 82.6^b^	39, 79.6^b^	<0.001
Body mass index (kg/m^2^)	31.1 ± 6.3	32.5 ± 7.2^a^	31.2 ± 5.4^a^	28.3 ± 4.3^b^	0.001
Smoking (n, %)	93, 50.0	52, 57.1	19, 41.3	22, 44.9	0.153
Hypertension (n, %)	154, 82.8	81, 89.0^a^	38, 82.6^a,b^	35, 71.4^b^	0.032
Serum creatinine (μmol/L)	71 (59‐89)	66 (55‐82)^a^	73 (58‐88)^a^	89 (68‐123)^b^	<0.001
eGFR (mL/min/1.73 m^2^)	91 (74‐102)	93 (82‐102)^a^	92 (81‐103)^a^	76 (49‐92)^b^	0.001
Total cholesterol (mmol/L)	4.6 ± 1.1	4.6 ± 1.0	4.7 ± 1.4	4.5 ± 0.9	0.786
HDL cholesterol (mmol/L)	1.18 ± 0.34	1.17 ± 0.30	1.17 ± 0.40	1.21 ± 0.35	0.683
Triglycerides (mmol/L)	1.6 (1.2‐2.4)	1.8 (1.2‐2.6)	1.6 (1.2‐2.2)	1.5 (1.2‐2.0)	0.519

Data are shown as mean ± SD, median (25th‐75th percentiles) or absolute frequency and percentage.

SBP, systolic blood pressure; DBP, diastolic blood pressure; eGFR, estimated glomerular filtration rate; HDL, high‐density lipoprotein.

Means, medians or percentages indicated with different letters were significantly different in the pairwise comparisons after correction for multiple testing (*P* < 0.05, Tukey or Dunn test for continuous variables and Bonferroni correction for categorical variables).

### 3.2 Comparison of miRNA levels between study groups

Plasma levels of miR‐29b and miR‐200b in blood donors and patients with type 2 diabetes are shown in Table 2. Expression of miR‐29b was detected in all study subjects, whereas miR‐200b was undetectable in seven of 186 (3.8%) type 2 diabetic patients. Patients with type 2 diabetes without DR had approximately twofold lower levels of miR‐200b in comparison to blood donors, while the levels of miR‐29b were not significantly different between them.

**Table 2:** MiRNA plasma levels in blood donors and type 2 diabetic patients

MiRNA	Blood donors (n = 20)	Without DR (n = 91)	*P* value^b^	NPDR (n = 46)	PDR (n = 49)	*P* value^c^
miR‐29b (log2 FC)	–1.79 (−2.14 to −1.19)	–1.16 (−2.32 to −0.35)	0.073	–1.66 (−2.89 to −0.27)	–1.91 (−2.54 to −1.23)^d^	0.022
miR‐200b (log2 FC)^a^	0.68 (0.10‐0.92)	–0.41 (−1.11 to 0.67)	0.001	–0.69 (−1.64 to 0.07)	–0.91 (−1.98 to 0.09)	0.078

Data are shown as median (25th**‐**75th percentiles).

FC, fold change.

a Data available for 179 type 2 diabetic patients (four patients without DR, one with NPDR and two with PDR had undetectable levels).

b Comparison of diabetic patients without DR and blood donors.

c Comparison of diabetic patients with PDR, NPDR and without DR.

d Significantly different from the group without DR after correction for pairwise multiple comparisons (*P* < 0.05, Tukey test).

**Figure 1:**
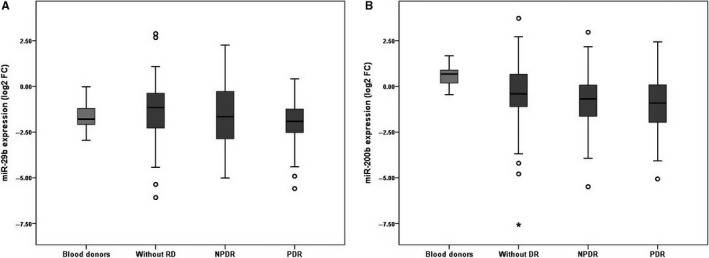
Comparative analysis of plasma levels of miR‐29b (A) and miR‐200b (B) in blood donors and type 2 diabetic patients according to the presence and severity of diabetic retinopathy. Expression levels are expressed as log2 fold change. A, *P* < 0.05 for the comparison of patients with proliferative DR and those without DR after correction for multiple testing (Tukey test). B, *P* < 0.05 for the comparison of diabetic patients without DR and blood donors (Mann‐Whitney test)

With regard to retinopathy, the mean levels of miR‐29b were 40% lower in patients with PDR as compared to those without DR. The same trend was observed for the miR‐200b, but the difference between the three groups of diabetic patients did not reach formal statistical significance (Table 2 and Figure 1).

### 3.3 Association analysis of miRNA levels with diabetic retinopathy

Univariate regression analysis showed that increased plasma levels of miR‐29b and miR‐200b were associated with a decreased risk of PDR (Table 3). As these two miRNAs were strongly correlated with each other in the group of subjects with type 2 diabetes (*r_s_* = 0.61, *P* < 0.001), we also used the logistic regression to test for their interaction (miR‐29b*miR‐200b). In this model, miR‐29 was associated with PDR (OR = 0.602, 95% CI: 0.407‐0.888, *P* = 0.011), but neither miR‐200b (OR = 0.828, 95% CI: 0.533‐1.287, *P* = 0.402) nor the interaction term (OR = 0.919, 95% CI: 0.802‐1.052, *P* = 0.219) was associated with PDR. However, miR‐29b levels did not remain associated with PDR after adjusting for the demographic and clinical variables that were also associated with this outcome in the univariate analyses (Table 3).

**Table 3:** Univariate and multiple logistic regression analysis for the association between miRNA levels and PDR

Variable	Univariate model		Multivariate model	
	OR (95% CI)	*P* value	OR (95% CI)	*P* value
miR‐29b (log2 FC)	0.694 (0.535‐0.900)	0.006	0.720 (0.420‐1.233)	0.231
miR‐200b (log2 FC)	0.797 (0.637‐0.997)	0.047	1.166 (0.757‐1.796)	0.486
Male gender	3.211 (1.560‐6.609)	0.002	1.231 (0.410‐3.698)	0.711
Age (y)	1.060 (1.011‐1.112)	0.016	1.038 (0.952‐1.131)	0.401
Diabetes duration (y)	1.077 (1.029‐1.127)	0.001	1.022 (0.950‐1.100)	0.557
Hypertension (yes/no)	0.309 (0.125‐0.762)	0.011	0.312 (0.075‐1.293)	0.108
Body mass index (kg/m^2^)	0.865 (0.793‐0.943)	0.001	0.887 (0.794‐0.992)	0.035
Insulin use (yes/no)	3.494 (1.558‐7.834)	0.002	3.461 (0.882‐13.589)	0.075
eGFR (mL/min/1.73 m^2^)	0.965 (0.948‐0.982)	<0.001	0.966 (0.942‐0.991)	0.007

OR, odds ratio; CI, confidence interval; FC, fold change; eGFR, estimated glomerular filtration rate.

## 4 DISCUSSION

This case‐control study assessed the association of the plasma levels of miR‐29b and miR‐200b with DR in type 2 diabetic outpatients from a tertiary university hospital in Southern Brazil. We reported, for the first time to our knowledge, that patients with PDR have lower plasma levels of miR‐29b as compared to those without DR. However, this association was lost after controlling for gender, age, diabetes duration, hypertension, BMI, insulin use and renal function. Although our study was not designed to investigate the association of miRNAs with type 2 diabetes and we cannot exclude the possibility that some blood donors have prediabetes or even diabetes, we found that diabetic patients had lower plasma levels of miR‐200b than blood donors.

In relation to miR‐29b and type 2 diabetes, our results are in accordance with a recent meta‐analysis that identified stress‐related miRNA biomarkers in different tissue types and species. The authors found that miR‐29b was not dysregulated in type 2 diabetes in the subgroup analyses of miRNAs in circulating blood and in human profiling studies.^14^ However, these findings contrast with studies that reported either higher circulating levels of miR‐29 in subjects with prediabetes^37^ and type 2 diabetes^35^, ^37^ or lower miR‐29b levels in those with prediabetes^38^ and who developed type 2 diabetes in the prospective population‐based Bruneck study.^39^ It is tempting to speculate whether miR‐29b is up‐regulated in type 2 diabetes as a failed attempt to compensate for the endothelial dysfunction, as evidenced by Widlansky et  al.^27^ The authors showed that miR‐29 is required for normal endothelial function and can restore it in cardiometabolic disorders.^27^ In addition, evidence obtained in knockout mice revealed that the miR‐29 family has a protective role in beta cells and a pathogenic function in the liver.^40^ Paradoxically, increased miR‐29 expression was reported to lead to increased insulin resistance, and decreased insulin secretion and signaling in metabolic tissues.^12^

With respect to miR‐29b and DR, our findings are in line with experimental studies of cultured retinal cells and diabetic murine models that showed that high glucose (HG) induces apoptosis and down‐regulates the expression of miR‐29b.^41^, ^42^ Expression of miR‐29b was decreased by up to 80% in rat retinal Müller cells treated with HG^41^, ^42^, ^43^ and by up to 60% over months in retina of diabetic rats compared to their normal glucose controls.^41^, ^43^ Overexpression of miR‐29b reversed the effects of HG by decreasing the expression of biomarkers of response to retinal injury and inflammation, such as glial fibrillary acidic protein and vascular endothelial growth factor (VEGF).^43^ In addition, transfection of miR‐29 in rat retinal Müller cells inhibited apoptosis,^41^ while the knockdown of miR‐29 promoted it.^41^, ^42^ In a zebrafish model of optic nerve crush injury, miR‐29b was overexpressed 3 days after the injury, suggesting that miR‐29b may protect retinal ganglion cells against cell death, thus regulating optic nerve regeneration.^26^ Experimental studies on other diabetic complications have found that overexpression of miR‐29b reduces apoptosis, protects primary isolated dorsal root ganglia neurons from streptozotocin (STZ)‐induced diabetic rats,^44^ and inhibits progressive renal inflammation and fibrosis in type 2 diabetes in db/db mice.^45^

Predicted and/or validated gene targets of miR‐29b include those associated with cell/survival apoptosis, extracellular matrix‐cytoskeleton signaling and DNA modification. Besides extracellular matrix constituents, collagen and intermediate filaments of the cytoskeleton, miR‐29b is also predicted to target genes involved with DNA (de)methylation and histone demethylation.^26^ In fact, histone methylation and acetylation are the two major mechanisms by which histone modification affects DR development.^46^ In retina, miR‐29b is expressed in the ganglion cells and the inner nuclear layer of normal and STZ‐induced diabetic rats.^47^ Taken together, these evidences indicate that down‐regulation of miR‐29b contribute to the progression of DR. In our study, levels of miR‐29b were 40% lower in patients with PDR as compared to those without DR.

In accordance with previous clinical studies,^15^, ^16^ we observed that the plasma levels of miR‐200b were decreased in patients with type 2 diabetes and even more reduced among those with PDR. In a previous study in Germany, plasma levels of miR‐200b were decreased at least threefold in type 2 diabetic inpatients in comparison to healthy controls.^15^ In a Chinese study, patients with DR (and diabetes type not reported) had about 60% lower plasma levels of miR‐200b than subjects without diabetes.^16^ In the retinas of non‐diabetic rats and diabetic patients, miR‐200b was found to be located in the vascular endothelium and in neuronal and glial elements.^48^ Studies in vitro showed that miR‐200b is highly expressed in normal human retinal endothelial cells (hRECs), while it was down‐regulated under HG environment.^49^, ^50^ Cell transfection with a miR‐200b mimic suppressed the migration of murine endothelial cells^51^ and the hRECs dysfunction by decreasing the levels of VEGF and transforming growth factor beta 1 (TGFB1),^49^ and by inhibiting vasohibin‐2, a protein that promotes the proliferation and migration of endothelial cells.^52^

Studies on animal models had also already shown the protective effect of miR‐200b on retinal diseases by inhibiting intraretinal neovascularization^16^, ^48^, ^51^, ^53^ and endothelial to mesenchymal transition (EndMT) in diabetic retina.^54^ McArthur et  al^48^ reported that miR‐200b was down‐regulated in retinas of STZ‐induced diabetic rats and in two endothelial cell lines incubated in HG. Transfection of endothelial cells and intravitreal injection of miR‐200b mimic prevented diabetes‐induced up‐regulation of VEGF and p300, a histone acetylator and a transcriptional coactivator. Increased endothelial permeability and angiogenesis were also prevented, and similar functional alterations were seen in the human retina from enucleated eyes obtained from patients with diabetes.^48^ In another study of the same research group, HG‐induced EndMT caused a reduction in miR‐200b (>30%) and the up‐regulation of VEGF and p300 in cultured hRECs. Such alterations were prevented in transfected cells with miR‐200b mimic and in transgenic mice with endothelial‐specific overexpression of miR‐200b.^54^ Using a model of retinal vasculopathy characterized by photoreceptor degeneration, vascular leak and deep retinal neovascularization in transgenic mice, Chung et  al^53^ observed that miR‐200b was down‐regulated 3 months after induced Müller cell disruption. Moreover, intravitreal injection of a miR‐200b mimic inhibited the established vascular leak from mild vascular lesions, whereas the anti‐miR‐200b promoted it. Similarly, STZ‐induced diabetic rats that were injected with miR‐200b mimic in the vitreous cavity showed less circuitous blood vessel, retinal microvessel density and endothelial cell nucleus, and had lower expressions of VEGFA and TGFB1 than the normal control group. The authors demonstrated experimentally that miR‐200b might alleviate DR development by down‐regulating its target gene *VEGFA*.^16^ Similar findings were obtained in Akita mice (a genetic model of diabetes) at 3 months after intravitreal injection of miR‐200b.^51^

During the course of this study, other investigators attempted to identify dysregulated circulating miRNAs in DR in humans.^17^, ^18^, ^19^, ^20^, ^21^, ^22^, ^23^ These studies were carried out in patients with type 1 diabetes,^17^, ^18^ type 2 diabetes^19^, ^20^, ^21^, ^22^ or diabetes type not reported,^23^ using serum,^18^, ^20^, ^22^, ^23^ plasma^19^, ^21^ or endothelial progenitor cells cultured from isolated peripheral blood mononuclear cells.^17^ They reported that angiogenic miRNAs are associated with incident DR,^18^ DR,^21^ NPDR^19^, ^22^ and PDR.^19^, ^23^ However, neither miR‐29b nor miR‐200b was investigated. Expression of either miR‐29b or miR‐200b was evaluated in studies in which patients with proliferative retinopathy were compared to non‐diabetic controls.^24^, ^25^, ^55^ Vitreous levels of miR‐29b did not differ between PDR patients (with diabetes type not reported) and subjects with macular hole, a non‐proliferative and non‐inflammatory disorder. Moreover, miR‐29b was not detected in the serum samples from the same patients.^24^ Plasma miR‐200b was up‐regulated in premature infants with retinopathy of prematurity stage 3 with plus disease, a proliferative vitreoretinopathy characterized by retinal ischaemia and neovascularization,^55^ while vitreous miR‐200b was increased in patients with PDR (41% type 2 diabetes) as compared to subjects with idiopathic macular holes.^25^

As a result of its observational and retrospective design, this study cannot determine whether the decreased levels of miR‐200b are causally related to type 2 diabetes or a consequence of it. The same is true for the association of decreased levels of miR‐29b and miR‐200b with PDR. As these miRNAs are not retina‐specific, we cannot determine their source and functional significance. In addition, the selection of the two miRNAs investigated in our study was based on intellectual choice instead of being based on gene expression profiling. By the time this study was designed, there were no clinical reports on miRNAs in DR in humans. Thus, we chose to study miR‐29b and miR‐200b based on the evidence from experimental studies showing their effect on apoptosis, fibrosis and angiogenesis, as DR was the primary outcome of our study. Despite the limitations, our findings support the perspective of using circulating miRNA levels as biomarkers of prognosis in type 2 diabetes and highlight the need for further research on them. As already recognized by other authors, although in vitro and animal studies are essential to elucidate the mechanistic effects, the same miRNAs may be differentially expressed between humans and animals,^14^ and even in different cell and tissue types, including vitreous, retinal endothelial cell and whole retina.^14^, ^25^

In conclusion, plasma levels of miR‐29b and miR‐200b were lower in patients with PDR as compared to those without DR. Considering that DR is a neurodegenerative disorder, down‐regulation of miR‐29b and miR‐200b could promote apoptosis, inflammation and disruption of Müller cell, thus leading to retinal degeneration, blood‐retinal barrier breakdown and angiogenesis. Moreover, plasma levels of miR‐200b were lower in patients with type 2 diabetes as compared to blood donors, indicating that miR‐200b may also be associated with type 2 diabetes itself. Therefore, our findings corroborate previous studies showing that miR‐29b and miR‐200b are dysregulated in the pathogenesis of type 2 diabetes and/or DR, supporting the need of further studies to evaluate their usefulness as prognostic biomarkers.

## CONFLICT OF INTEREST

The authors confirm that there are no conflicts of interest.

## AUTHOR CONTRIBUTIONS

MEDCS and KGS conceived the study design and performed the statistical analyses. ERP and RCS enrolled the patients and updated the database. ERP enrolled the blood donors, performed the eye fundus examination and the RT‐qPCR experiments. DL and FM revised the retinography images to confirm the diagnosis of retinopathy. LHC contributed to acquire data from patients and supervised the clinical stages of the study. DC and NCM designed the RT‐qPCR experiments. DC and KGS contributed funding resources. KGS supervised the study, interpreted the data and wrote the paper. All authors reviewed the manuscript and approved the submitted version.
